# Modelling Nuclear Morphology and Shape Transformation: A Review

**DOI:** 10.3390/membranes11070540

**Published:** 2021-07-16

**Authors:** Chao Fang, Jiaxing Yao, Xingyu Xia, Yuan Lin

**Affiliations:** 1 Department of Mechanical Engineering, The University of Hong Kong, Hong Kong, China; chfong@connect.hku.hk (C.F.); yaojx@connect.hku.hk (J.Y.); u3005822@connect.hku.hk (X.X.); 2 HKU-Shenzhen Institute of Research and Innovation (HKU-SIRI), Shenzhen 518057, China; 3 Advanced Biomedical Instrumentation Centre, Hong Kong Science Park, Shatin, New Territories, Hong Kong, China

**Keywords:** nuclear mechanics, shape transformation, continuum models

## Abstract

As one of the most important cellular compartments, the nucleus contains genetic materials and separates them from the cytoplasm with the nuclear envelope (NE), a thin membrane that is susceptible to deformations caused by intracellular forces. Interestingly, accumulating evidence has also indicated that the morphology change of NE is tightly related to nuclear mechanotransduction and the pathogenesis of diseases such as cancer and Hutchinson–Gilford Progeria Syndrome. Theoretically, with the help of well-designed experiments, significant progress has been made in understanding the physical mechanisms behind nuclear shape transformation in different cellular processes as well as its biological implications. Here, we review different continuum-level (i.e., energy minimization, boundary integral and finite element-based) approaches that have been developed to predict the morphology and shape change of the cell nucleus. Essential gradients, relative advantages and limitations of each model will be discussed in detail, with the hope of sparking a greater research interest in this important topic in the future.

## 1. Introduction

As one of the most important cellular components, the nucleus is widely believed to play critical roles in processes such as mitosis [1,2,3], cell spreading [4,5,6,7] and migration [8,9,10,11,12]. For instance, the nuclear membrane in most eukaryotic cells will be dissolved during mitosis and then get reassembled in daughter cells [13,14] while it will remain largely intact in fission yeasts, i.e., only local disassembly of the nuclear envelope (NE) occurs at the end of mitosis to accomplish daughter nuclei separation [15]. The role of the nucleus as a mechanosensor for environmental stimuli [16,17,18,19], a transducer for downstream signaling [20,21,22,23,24] and a ruler for active cellular responses [10,11,25,26] has also been well-documented. Taking cell migration as an example, severe distortion could take place when the cell passes through tight spaces or moves in blood vessels. In plant cells, turgor pressure in the vacuole will also deform other organelles [27,28]. Such deformations can be transduced to the nucleus, lead to its morphology changes and eventually trigger corresponding cell reactions. Finally, the nucleus has been examined as a potential marker for diseases like cancer [22,29,30] and laminopathies, including Hutchinson–Gilford Progeria Syndrome [22,31,32,33,34,35,36] and Emery–Dreifuss muscular dystrophy [37,38,39], where the disorder was found to arise from the absence/malfunction of gene *LMNA* (coding lamin A/C) or *STA* (coding nuclear protein emerin) that leads to nuclear dysfunction and morphology abnormalities.

Various advanced techniques (such as microrheology [40,41], micropipette aspiration [42,43,44,45], microplate manipulation [46,47], atomic force microscopy (AFM) indentation [48,49], fluorescence correlation spectroscopy (FCS) [50], all-optical Brillouin microscopy [51], optical tweezer [52], laser microsurgery [53]) have been developed/adopted in the past two decades to monitor nuclear morphology change or characterize the physical properties of individual nuclear components. However, due to complicated interactions/connections among different nuclear constituents, it’s not easy to experimentally decouple their roles in determining the shape and bulk mechanical response of the nucleus. For this reason, many theoretical models have been developed to delineate the individual and collective contributions of each nuclear constituent [54,55], as well as provide explanations to a variety of puzzling observations [56,57].

Roughly speaking, existing models capable of describing/predicting nuclear morphology transformation can be divided into two categories: continuum and molecular dynamics models. Here, we focus on continuum-level approaches while molecular dynamics simulations will not be discussed. Interested readers could refer to [58] for a review on that front. Specifically, different biophysical elements involved in regulating nuclear shape change will be discussed first before the introduction of three main types of continuum (i.e., energy minimization, boundary integral and finite element-based) models. Essential gradients, advantages and suitability (for examining different cellular processes) of each approach will be discussed in detail. In the end, we will also share our views on the future of this important field.

## 2. Biophysical Elements Involved in Regulating Nuclear Morphology

Before we plunge into the details of different models, it is important to identify major biophysical elements involved in nuclear morphology regulation. For example, both the physical characteristics of the surrounding environment and the complex intracellular/intranuclear structure were found to play key roles in the mechanotransduction and shape transformation of the cell nucleus. Therefore, the influencing biophysical factors can be approximately summarized as nuclear structure, cytoskeleton (i.e., actin filaments, intermediate filaments and microtubules), extracellular matrix (ECM), cell adhesion, spatial physical confinement and medium osmolarity.

### 2.1. Internal Structure of the Nucleus

**Figure 1:**
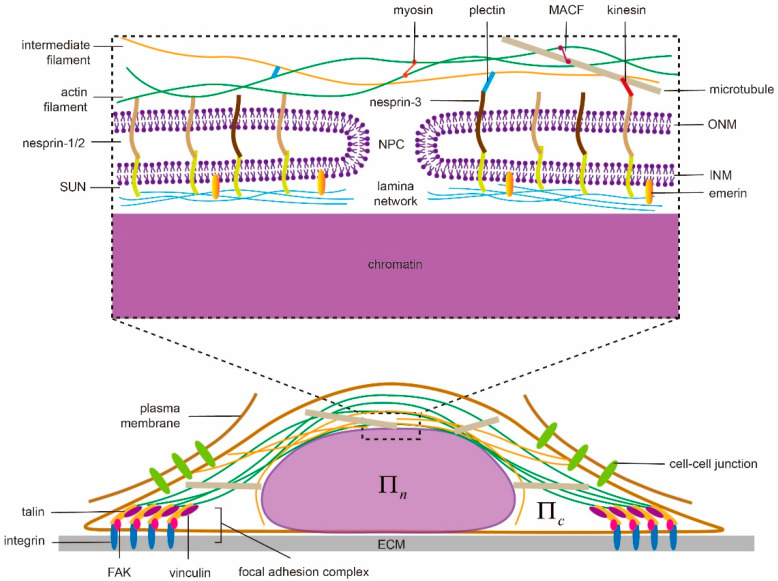
Illustration of biophysical elements regulating nuclear morphology and shape transformation in adherent cells, i.e., nuclear structures and connections, cytoskeleton, extracellular matrix (ECM), cell adhesions and osmolarity. ECM can modulate the nuclear shape through focal adhesions and the associated stressed cytoskeleton with patterns known as perinuclear actin cap and vimentin cage. They transduce inter/intracellular contraction generated by motor proteins to compress the nucleus whose proper functioning largely depends on the linkers of nucleoskeleton and cytoskeleton (LINC) complexes (composed of nesprin family proteins in outer nuclear membrane (ONM) and Sad1/UNC-84 (SUN) proteins in inner nuclear membrane (INM)) and active motor proteins. In addition to chromatin condensation, the integrity of the lamina network layer and its links to INM (via emerin) defines the nuclear deformation ability. Any malfunction of them could lead to abnormal nuclear morphology or failure of shape transformation. In contrast, the osmotic pressure difference between the nucleoplasm ($\Pi_{n}$) and cytoplasm ($\Pi_{c}$) could tune the shape by water absorption/leakage without solid contact.

Serving as the space for protecting genetic materials and the site for gene transcription, the cell nucleus is known for its complicated inner organization. From the structure point of view, the nucleus contains a nuclear envelope (NE), a lamina layer underneath the NE and chromatins enclosed in the center (Figure 1). The NE consists of double bilayer membranes—an inner nuclear membrane (INM) and an outer nuclear membrane (ONM) [59]. These two membranes are separated by a 30–50 nm-thick interval [23] in general but can fuse together locally to form nuclear pore complexes (NPCs) on the NE [60,61,62], allowing transport of small size solutes, water molecules, proteins, mRNA and macromolecular complexes between the cytoplasm and nucleoplasm to take place. Underneath the INM, a lamina layer (a complex network of intermediate filaments lamin A/C, B1 and B2), with thickness ranging 10–50 nm [63] is usually formed in mammalian cells but is absent in fission yeasts [64], making their nuclei more susceptible to force-induced shape changes. This lamina layer binds to INM via proteins such as emerin while, at the same time, connects to the cytoskeleton through the so-called linkers of nucleoskeleton and cytoskeleton (LINC) complex (composed of the nesprin family proteins, that include Klarsicht/ANC-1/SYNE homology (KASH) domain, in ONM and Sad1/UNC-84 (SUN) proteins in INM) [23,65,66]. On the other hand, through the lamin-associated proteins (LAP), the lamina network also establishes connections with chromatins [67] whose adjustable condensation/decompaction can then affect gene expression of the cell [68,69]. This “LINC-NE-lamina layer-chromatin” chain serves as a critical pathway for transmitting extranuclear signals to the nucleus and eventually allowing mechanotransduction in cells to take place.

### 2.2. Cytoskeleton

By transducing forces generated by motor proteins like kinesin, dynein and myosin or pushing the nuclear membrane directly via polymerization, the cytoskeleton (composed of actin filaments, intermediate filaments and microtubules) plays important roles in regulating the shape of the cell nucleus. Specifically, F-actin can connect directly to the actin-binding domain of nesprin-1/2 [70] on the nuclear membrane while the binding between microtubules and nesprin-1/2 has been suggested to be mediated by kinesin [71,72]. In contrast, cytoskeleton linker protein plectin is believed to link intermediate filaments to nesprin-3 on the ONM [73] (Figure 1). Note that the three categories of filaments are interconnected with each other via cross-linkers as well. For instance, microtubules-actin cross-linking factor (MACF) works as a joint between F-actin and microtubules [74] while plectin connects actin and intermediate filaments [75]. As such, the presence or absence of any type of these filaments could adjust the mechanical response of the cytoskeleton and eventually regulate nuclear morphology. In Drosophila, for example, polymerizing microtubules can indent the nucleus, cause its movement [76] and induce membrane grooves [77]. Interestingly, the grooves may even develop into full membrane folds [78]. In another case, when the cell is detached from the substrate, microtubules and the perinuclear actin cap can compress the nucleus and lead to buckling of the NE [79]. Note that, perinuclear actin cap is an organized doom-like stress fibers network, connecting to LINC complexes (refer to Figure 1), that has been observed in a wide range of adherent cells (i.e., 3T3 fibroblasts, C2C12 mouse myoblasts, human endothelial cells and human ovarian epithelial cells) [7]. Such structure is believed to play critical roles in protecting the nucleus from extracellular physical disturbances [4], regulating cell migration [80] and facilitating mechanosensation/mechanotransduction [81]. For instance, during the spreading of cells, the apical actin cap will transmit cortical tension to compress the nucleus. Then the flattened nucleus will be resisted/confined by lateral stress fibers, eventually resulting in its elongation [57]. Similarly, it was found that vimentin, a type III intermediate filament protein, could form a cage-like network around the nucleus, which organizes its shape and helps it resist severe deformations by enhancing perinuclear stiffness [82,83]. Experimentally, the nuclei of vimentin deficient (vim^−^) cells exhibit a clearly rounder morphology compared to the oblate spheroidal nuclei in vim^+^ cells [82]. On the other hand, the nuclear contour of the latter is much smoother than that in vimentin deficient cells where folding and invaginations of the nuclear membrane are often observed [82,84]. Physically, it is believed that the elevated tension level within the nuclear membrane, caused by the surrounding vimentin cage, could suppress its thermal fluctuations and lead to a smoother morphology [82].

### 2.3. Extracellular Matrix and Cell Adhesion

It has been well documented that the cell can form larger focal adhesions and develop higher intracellular contraction on a more rigid extracellular matrix [85,86], suggesting cells are capable of sensing the physical characteristics of their microenvironment and then reacting accordingly [86,87,88,89,90,91]. Recent evidence also indicated that the nuclear shape of cells is regulated by ECM rigidity as well. Specifically, the nucleus was found to be flattened on rigid substrates while remained tall on soft ones. Interestingly, such influence (of ECM rigidity) disappears once myosin activity is inhibited or LINC complexes in the cells are disrupted [6]. Furthermore, ECM geometry also appears to play a role in affecting the shape of the cell [92] and its nucleus [7,57]. In particular, ECMs with high aspect ratios were found to result in severely elongated nuclei along with anisotropically distributed focal adhesions [57]. It must be pointed out that these aforementioned nuclear shape changes are all mediated by cell-ECM adhesions (Figure 1) where a number of proteins, including integrin, vinculin, focal adhesion kinase (FAK), and talin, are assembled together [93]. For example, the ECM rigidity sensing capability of cells was thought to originate from the force-dependent unfolding (and subsequent vinculin binding) of talin [86]. Interestingly, recent evidence also suggested that cadherin-based cell-cell contact (Figure 1) could affect the positioning [94] and deformability [95] of the cell nucleus as well.

### 2.4. Physical Confinement

A myriad of investigations showed that, when passing through tightly confined spaces, the cell nucleus can be squeezed into an elongated or a dumbbell-like shape [8,10,11,12,96,97,98]. Furthermore, when the confinement is narrower than a threshold size, unfolding of the NE (i.e., flattening of the originally wrinkled surface) will take place, releases calcium from the nuclear membrane and activates the calcium-dependent phospholipase cPLA2, eventually enhancing the actomyosin contractility and migration capability of the cell [10,11]. Actually, this has been thought of as a self-protection mechanism for cells to escape tight confinements (and hence avoid possible damages induced by severe cellular deformations). Note that, unlike the NE flattening due to integrin-based adhesion or cytoskeleton compression [4], the unfolding of NE, in this case, is merely caused by physical confinements.

### 2.5. Osmolarity

In contrast to all other factors mentioned above, no physical contact is needed for osmotic pressure to regulate the shape and activity of cells [99,100,101,102,103]. Interestingly, although NPCs allow small-sized molecules and water to pass through the nuclear membrane, the volume and shape of the cell nucleus are still sensitive to extracellular osmolarity. For example, the nuclear volume was observed to grow linearly with the decreasing medium osmolarity initially but become rather insensitive to it in the deep hypo-osmotic range while the nuclear surface becomes smoother [104]. In addition, after the sudden removal of mechanical stresses exerted on the nucleus, the rapidly changed osmotic pressure was found to lead to shrinkage of the nucleus as well as buckling of the NE [79].

## 3. Continuum Models for Describing Nuclear Morphology

### 3.1. Energy Minimization Model

After the pioneering works by Canham [105] and Helfrich [106], the approach of energy minimization has been widely adopted in describing the shape change of biological membranes or fluid vesicles including the NE [107,108,109,110,111,112]. Essentially, the stable shape of NE under loading is the one that minimizes the total energy of the system. Specifically, by taking membrane bending and stretching into account, this elastic energy stored can be expressed as

(1) $$W=\overset{}{\underset{S}{\iint }}[\frac{1}{2}K_{b}{\left(C_{1}+C_{2}\right)}^{2}+\frac{1}{2}K_{A}\left(\frac{A-A_{0}}{A_{0}}{)}^{2}\right]\;dS$$

where $K_{b}$ refers to bending rigidity of membrane, $C_{1}$ and $C_{2}$ are the two principal curvatures. Note that the so-called spontaneous curvature of membrane and Gaussian bending energy are ignored here because the former vanishes when the lipid composition of bilayer membrane is the same and the integration of the Gaussian curvature on a closed surface is simply a constant. The second term of the integrand represents stretching energy density in which the membrane tension can be expressed as $\gamma =K_{A}\frac{A-A_{0}}{A_{0}}$, with $K_{A}$, A, $A_{0}$ being areal expansion modulus, real and un-stretched membrane area, respectively. Sometimes, γ is treated as a constant (i.e., independent of membrane area A) because the ONM is continuous with the endoplasmic reticulum (ER) which works like a lipid reservoir to maintain the membrane tension level [107,108,109].

**Figure 2:**
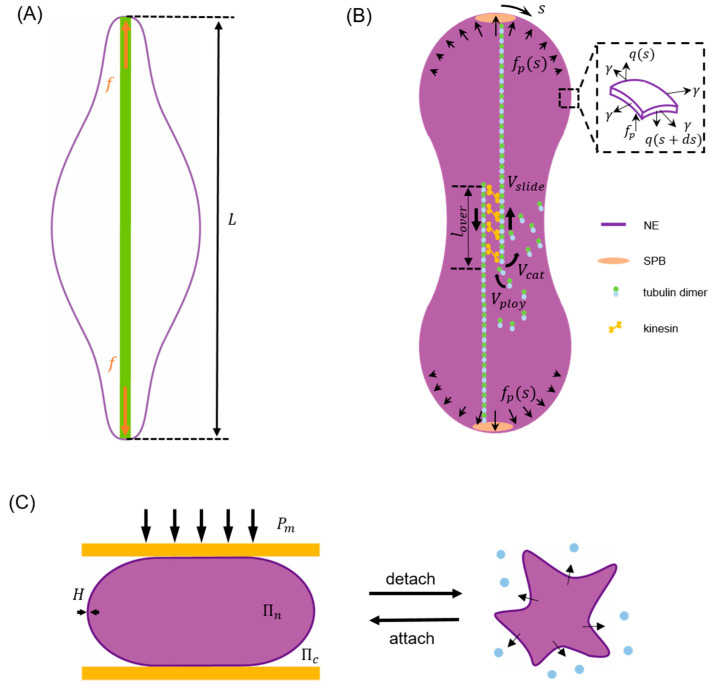
Schematics illustrating typical examples of energy minimization model, boundary integral model and finite element-based model for describing the nuclear envelope (NE) shape change in different cell processes. (**A**) As the driving force term of closed mitosis in the energy minimization model, a concentrated poleward force f generated by elongating microtubule bundles (green) pushes the two poles of NE to a separation given by L [109]. (**B**) In the boundary integral model for closed mitosis [117], the NE deformation is assumed to be driven by distributed poleward force $f_{p}$ and resisted by membrane elasticity and viscous drag. Inset shows the forces (membrane tension γ, transverse shear q and poleward force $f_{p}$) acting on the deformed envelope. Here, s represents the arc length coordinate with s=0 at the top pole. Microtubule bundles are assumed to have an overlap length $l_{over}$ where attached kinesin motors can generate forces to slide antiparallel microtubules with a velocity $V_{slide}$ to effectively push the NE. Meanwhile, the polymerization (with rate $V_{poly}$) and catastrophe-induced disassembly (with rate $V_{cat}$) of microtubules are allowed to take place on their plus ends in the center. (**C**) In the finite element method (FEM) model describing NE buckling during cell detachment, the perinuclear actin cap and surrounding microtubules are simplified as a compressive plate (orange) and a compression ($P_{m}$), respectively. As the detachment goes on, increment of $P_{m}$ results in water (blue dots) efflux from the nucleus, an abrupt increase in the osmotic pressure difference between the nucleoplasm ($\Pi_{n}$) and cytoplasm ($\Pi_{c}$), and eventually the buckling of NE shell with thickness H [79].

Taking closed mitosis of fission yeast as an example, since the volume enclosed by the NE $\left(V_{n}\right)$ remains largely as a constant [2,3] during this process a term $–PV_{n}$ needs to be added to the right hand side of Equation (1) with P working as a Lagrange multiplier [107,109] to enforce the volume conservation condition. In addition, given that gradual separation between its two poles is driven by growing spindle microtubules inside the nucleus during closed mitosis [1,2,3,53], a corresponding potential term should also be considered. For instance, by assuming that the elongating microtubule bundles effectively generate a concentrated force f on both poles (Figure 2A), a term

(2) $$W_{mt}=–fL$$

can be added to Equation (1) with L representing the pole-to-pole distance of the NE [109].

This simple description successfully explained the formation of single or double lipid tethers after the connection between microtubule bundles and spindle pole bodies (SPBs) was destroyed by laser severing [1]. On the other hand, the fact that chromatins bind to INM via LEM (LAP2, emerin, Man1) domain proteins, i.e., Heh1, Heh2 [113,114], suggests that the spindle force could be transmitted to the NE through chromatins. Therefore, Zhu and coworkers proposed that the pushing force f from growing microtubules is distributed to the NE over a load transmitting area with characteristic size $s_{c}$. Effectively, a potential term of the form [107]

(3) $$W_{mt}=-\int_{0}^{s_{1}}2\pi rf\left(s\right)zds$$

was added to Equation (1) where $s_{1}$ is total arc length between the apex and nadir of the NE while r and z corresponding to the abscissa and ordinate of membrane within the load transmission region [107]. Variations of $s_{c}$ were found to result in the appearance of complex nuclear shapes including tethers, pear, spherical cylinder and dumbbell, in agreement with experimental observations [1,2,3,107]. Following a similar approach, Castagnetti and coworkers examined how the deformability of chromosomes influences the morphology of dividing NE [108]. Specifically, the chromosome was treated as a straight bar bent into a circular arc with curvature C, leading to bending energy of chromosomes as

(4) $$W_{chro}=\frac{1}{2}k_{chro}\int^{\;}C^{2}ds$$

with $k_{chro}$ representing the bending rigidity of a chromosome. Note that, in this scene, it was assumed that chromosomes contact with the NE only at two ends (of the bar). It must be pointed out that, besides being widely adopted in examining the morphology of cells or nuclei, the energy minimization approach has also been used in studying problems such as the distribution of NPCs, connecting cytoplasm to the nucleoplasm, on the nuclear membrane [115,116].

### 3.2. Boundary Integral Model

The boundary integral method is well known for its low computation cost due to the reduction in calculation dimensionality (with the use of fundamental solutions) and its efficiency in examining problems like boundary tracking. Since NE serves as a physical barrier to separate cytoplasm from nucleoplasm (both can be viewed as fluid-like or solid-like [56,117] media), it is not surprising to see that boundary integral method has been used to describe its shape evolution. For example, a model was developed recently to examine how the viscous response of cytoplasm [118,119,120] and nucleoplasm [40,50,121,122,123] influences the nuclear morphology change during closed mitosis (refer to Figure 2B). Since the Reynolds number involved is small (i.e., Re << 1) in this case, the Green’s function of Stokes flow can be utilized to express the velocity, along the i-th direction, of an arbitrary point ξ on the NE surface $S_{y}$ as [124,125]

(5) $$u_{i}\left(\xi \right)=\iint_{S}^{}u_{i}^{j}\left(\xi ,y\right)n_{j}\left(y\right)f\left(y\right)dS_{y}$$

where $u_{i}^{j}\left(\xi ,y\right)=-\frac{1}{8\pi \eta }\left(\frac{\delta_{ij}}{r}+\frac{\left(\xi_{i}-y_{i}\right)\left(\xi_{j}-y_{j}\right)}{r^{3}}\right)$ (i,j=1,2,3) is the so-called Stokeslet with r=|ξ−y| and η being the viscosity of the nucleoplasm and cytoplasm, and f refers to the force acting on the NE along its normal (n) direction, consisting of (i) the poleward force $f_{p}$ generated by kinesin motors residing in the overlap region of microtubule bundles [126,127]; (ii) membrane stretching force $f_{t}$ following Young-Laplace law and (iii) bending-induced transverse shear $f_{b}$ inside the membrane [128], that is

(6) $$f\left(y\right)=f_{p}+f_{t}+f_{b}.$$

Furthermore, to clarify how kinesin motors and microtubule dynamics are coupled with the elastic deformation of NE and eventually dictate its macroscopic shape change in an explicit manner, classical Hill’s law was assumed to describe the relationship between microtubule sliding velocity and generated poleward force [129]. The model predicted that, starting from a sphere, the NE would undergo initial elongation, necking and final spindle poles separation to become a barbell at the end of closed mitosis while defects in microtubules resulting in an abnormal division of the nucleus as observed in experiments. In addition, it was found that the process is dominated by membrane stretching which absorbs ~90% of the work done by poleward force while the influence of viscosity is negligible.

In contrast, to capture the flattening of fibroblast nucleus during cell spreading, the mixture of cytoplasm and cytoskeleton was treated as a contractile compressible material confined by the plasma membrane and NE [56]. The constitutive relation, in this case, was proposed as

(7) $$\sigma =2\eta \dot{\varepsilon }+\sigma_{c}I$$

where σ is the stress tensor, η refers to effective viscosity of the material, $\dot{\varepsilon }$ means strain rate tensor, $\sigma_{c}$ is active contractile stress and I represents identity tensor. Meanwhile, four external force terms were considered: (1) nuclear resistance to volume compression/expansion, (2) nuclear resistance to surface area expansion, (3) plasma membrane tension and (4) cell-substrate friction due to actin retrograde flow. In addition, assembly of F-actins was assumed to take place with a rate $v_{a}$ at the cell boundary. Finally, with the help of Kelvin’s fundamental solutions for axisymmetric linear elasticity, the velocity field can be obtained by solving the momentum conservation equation ∇·σ=0 with the boundary integral method [56]. Interestingly, it was found F-actin assembly at the boundary alone is significant enough to drive the flattening of the cell nucleus while forces induced by microtubules, intermediate filaments, LINC complexes and myosin contraction were all dispensable.

### 3.3. Finite Element-Based Models

Another popular way to model nuclear morphology transformation is to treat the nucleoplasm as an incompressible fluid or a deformable solid enclosed by an elastic shell, representing the NE [49,51,54,55,79,97,98,130,131]. The finite element method can then be used to implement different constitutive descriptions of the material (in commercial software packages like ANSYS, ABAQUS and COMSOL Multiphysics) to capture the shape change of the nucleus.

An early trial along this direction was made by Varizi and Mofrad more than a decade ago where contributions from different components inside the nucleus on its deformation were examined [54,55]. Specifically, INM, ONM and lamina layer were treated as linear elastic shells with characteristic bending stiffness of the order of $10^{-16}\;\mathrm{mN}/m$ and stretching stiffness in the range of 1–10 mN/m. In contrast, the enclosed nucleoplasm was represented by a viscoelastic Maxwell material with a single characteristic relaxation time. Interestingly, it was found that the bending and stretching of NE result in a more diffused stress distribution within the nucleus under AFM indentation, eventually leading to a much reduced maximum effective (von Mises) stress than those predicted by models where the role of NE was neglected. In addition, the force-displacement response was shown to be more sensitive to the nucleoplasm modulus and the bending stiffness of NE than the properties of the lamina layer. Similarly, by treating the NE and lamina layer as an infinitely thin elastic shell wrapping around a compressible elastic solid [49], Hobson and coworkers reported that the nuclear response is governed by nucleoplasm elasticity at small indentation depth but becomes increasingly dominated by stretching of the nuclear membrane as indentation depth increases.

Recently, with the discovery of actin cap formed around the nucleus [7], its role in nuclear shape evolution has also been investigated. For example, to simulate the deformation of nucleus driven by actin polymerization in T cells, the actin layer and nucleus shell were both treated as isotropic compressible neo-Hookean materials [132] with the strain energy function given by

(8) $$W=\frac{1}{2}\mu \left(I_{1}-3\right)-\mu lnJ+\frac{1}{2}\lambda {\left(lnJ\right)}^{2}$$

where μ and λ represent the shear and bulk modulus, respectively. $I_{1}$ is the first invariant of the right Cauchy-Green deformation tensor and J=det(F) with F being the deformation gradient. It was found that, as actin polymerization progresses, the actin layer gradually engulfed the nucleus and then compressed it into an elongated shape. On the other hand, by describing the perinuclear actin cap as a rigid plate to confine the nucleus (refer to Figure 2C), the model was also used to explain the observed NE buckling during cell detachment [79]. In this case, the nuclear volume ($V_{n}$) was assumed to change according to the osmotic (i.e., ΔΠ which is inversely proportional to $V_{n}$) and hydrostatic (ΔP) pressure difference across the NE as

(9) $$\frac{dV_{n}}{dt}=\zeta \left(\Delta \Pi -\Delta P\right)$$

with ζ representing the water permeability of the membrane. Using this framework, Kim and coworkers showed that, when cell detachment occurs the aggregation of microtubules surrounding the nucleus results in an elevated hydrostatic pressure difference across the nuclear membrane, a water efflux from the nucleus and eventually the buckling of NE itself as observed in experiments [79]. In addition to describing the actin cap as a passive material, active stresses generated within the actin cytoskeleton have also been considered in modeling cell transendothelial migration [97,98], where the nucleus (pulled by actomyosin contraction generated at cell front [133,134]) must squeeze through extremely narrow gaps. Specifically, a chemo-mechanical model was introduced to account for the contractile stress (σ) as

(10) σ=ρ+Kε

where ε, K is the strain and effective passive stiffness of the actin filaments, and ρ refers to the force-dipole density (representing active myosin contraction) taking the form

(11) $$\rho =\frac{\beta \rho_{0}}{\beta -\alpha }+\frac{\alpha K-1}{\beta -\alpha }\varepsilon$$

with $\rho_{0}$ being the contractility in the absence of adhesions, and α and β representing mechano-chemical parameters that arising from the coupling (via Rho-associated protein kinase (ROCK) mediated phosphorylation) between the mechanical stretch of F-actins and the engagement/assembly of myosin motors [135].

Most recently, a more comprehensive model was proposed by Alisafaei and co-workers [131] where almost all biophysical regulators of nuclear shape, like the elastic response of chromatin and NE, anisotropic actomyosin contraction, actin polymerization, focal adhesion and ECM geometry, have been taken into account. In addition to adopting the conventional hyperelastic description of the NE (lamina layer included), i.e.,

(12) $$\sigma_{ij}=C_{ijkl}\varepsilon_{kl}=\left(C_{ijkl}^{\left(I\right)}+C_{ijkl}^{\left(F\right)}\right)\varepsilon_{kl}$$

with $\sigma_{ij}$ and $\varepsilon_{kl}$ being the stress and strain, $C_{ijkl}^{\left(I\right)}$ referring to the initial stiffness and $C_{ijkl}^{\left(F\right)}$ representing the tension stiffening effect of the lamina network, a similar but more complex mechano-chemical feedback description (compared to Equation (11)) was proposed for capturing the anisotropic actomyosin contraction in the cytoskeleton which was believed to stretch F-actins but compress microtubules, respectively. Interestingly, this model was shown to be capable of explaining a variety of experimental findings, including the ECM geometry regulated actin organization, focal adhesion-induced cytoskeletal alteration and nuclear morphology change [7,57], nuclear invagination [76,77,78], and cytoskeleton-regulated apparent modulus of the nucleus [51].

Finally, we want to emphasize two things. Firstly, the applications of the aforementioned models are far beyond tracking nuclear shape changes. For instance, finite element simulations were also widely used to extract the elastic [41], poroelastic [48,97], hyperelastic [46] or even plastic [97,130] characteristics of the cell nucleus under different experimental and physiological conditions. A typical simulation result revealed that the nucleus is an order of magnitude stiffer than the cytoplasm, consistent with experimental findings [40,42,136,137], and a softer lamina network leads to a higher irreversible deformation of the nucleus as reported in [45]. Secondly, many phenomenological models were not discussed here because they don’t fit into the three types of approaches discussed above. For instance, the nucleus has been treated as a standard linear viscoelastic [42] or Voigt-Maxwell [121,122] solid. A mechanical network and semi-analytical models were also proposed to describe nuclear blebbing [138,139] and compression of cell nucleus [140].

## 4. Conclusions and Outlooks

In this review, different theoretical approaches developed to model the nuclear shape transformation during various cellular processes were discussed. When compared with each other, the energy minimization approach can identify the steady-state shape of the NE in a simple and elegant manner, and hence has been widely used in situations where the deformation process is slow (and therefore quasi-static equilibrium of the nuclear membrane can always be assumed). On the other hand, the dynamic nature of the boundary integral approach makes it suitable for cases where the coupling between nucleoskeleton/cytoskeleton dynamics and NE deformation needs to be taken into account. Finally, unlike these two aforementioned approaches, the nonlinear response of the NE (such as its strain-stiffening behavior) can easily be incorporated in finite element-based models. In addition, different constitutive descriptions of the cytoplasm and/or nucleoplasm can also be introduced in finite element-based models (rather than simply treating it as a viscous fluid in boundary integral models or representing its effect as merely providing a volume conservation constraint in the energy minimization approach), allowing us to systematically examine how nuclear morphology transformation is influenced by the physical characteristics of these subcellular components.

Despite all the progress mentioned here, several important issues/challenges remain unsettled. First of all, more precise descriptions of the mechanical response of the nucleus and its components are needed. For instance, the nucleoplasm (including chromatins) has been conveniently modelled as a homogeneous elastic [49,108,131], viscoelastic [54,55], poroelastic [97] solid or a viscous fluid [79,117] in different studies. In reality, it is conceivable that deformation of the nuclear envelope will squeeze/distort the chromatins inside which can then in return affect the deformability of the nucleus as a whole. In addition, condensation of chromatin can also take place during the cell cycle and therefore alter the mechanical properties of the nucleus. More realistic models are needed in the future to take these important factors into account.

Furthermore, a unified model capable of incorporating detailed environmental/cellular/nuclear structures in influencing the nuclear morphology is still lacking. As can be seen from this review, the nucleus was often treated as a homogeneous body in the early years before the nuclear membrane was modelled separately from the nucleoplasm. After the discovery of the actin cap, descriptions of cytoskeleton started to appear in different models to highlight its important regulatory role. Recently, attempts of including focal adhesion and active mechano-chemical feedback into the model have also been made. In the future, given that most deformations of the nucleus were caused by forces generated within the cytoskeleton or nucleoskeleton, analyzing the interactions between nuclear membrane and growing/evolving bio-filaments will likely be another key for modeling NE shape change, similar to that in examining cell migration [141,142,143] and filopodium formation [144,145,146] where cellular movement is known to be driven by the polymerizing F-atcin network. Although microtubule dynamics and macroscopic deformation of nuclear membrane during closed mitosis were connected together in a simple manner by the boundary integral model proposed recently [117], a much more concentrated effort is needed to extend this to other processes.

Lastly, by revealing the biophysical mechanisms behind them, modelling works are expected to go beyond explaining observations to provide insights for the design of future experiments and biomedical applications. Actually, many assumptions made in the models discussed above came from and evolved with experimental observations. For example, the propelling force from separating microtubules during closed mitosis was regarded as a concentrated one initially [108,109]. As evidence emerged showing that chromosomes could bind to INM (and therefore presumably transmit load), such force was then modified to be distributed on the NE [107,117]. Similarly, linear elastic description of NE was adopted in the early days [55] which was then improved to be hyperelastic to reflect the widely observed strain-stiffening behavior of biological tissues [147,148,149]. On the other hand, modelling works have helped experimentalists to design new experiments or catch important phenomena that went unnoticed previously. For instance, Li and coworkers theoretically found spreading itself is enough to drive the nucleus flattening without the help of other factors [56]. This prediction then likely led Patteson and coworkers to carry out further experimental investigations to examine the correlation between the spreading level and nuclear sphericity in vim^+^/vim^−^ cells [82]. Another example is the predicted sudden necking of NE during closed mitosis, a phenomenon that often went unnoticed in dividing yeast cells before this prediction was reported [117]. On the application front, it is conceivable that combining modelling with machine learning could potentially lead to strategies for the diagnosis and prognosis of cancers and laminopathies [150,151] where the abnormal nuclear shape is realized as a disease marker [22]. This is certainly an important direction that warrants investigations in the future.

## Data Availability

Not applicable.
